# When Maxillofacial CBCT Permits Fortuitously to Diagnose Primary Non-Hodgkin’s Lymphoma: A Case Report

**DOI:** 10.5334/jbsr.3682

**Published:** 2024-09-05

**Authors:** Pierre-Louis Polard, Adrian Tempescul, Karen Vallaeys

**Affiliations:** 1 Bucco-Dental Medicine Resident, Brest University Hospital, Western Brittany University, France; 2 Hospital Practitioner in Hematology Department, University Hospital Center, Brest, France; 3 Associate Professor Hospital Practitioner in Odontology, Oral Medicine and Oral Surgery Department, University Hospital Center, University of Occidental Brittany, Brest, France; Laboratory of Medical Information Processing, UMR 1101, University Hospital Center, Brest, France

**Keywords:** intrabone malignant non-Hodgkin’s lymphoma, maxillary, lymphoma large B-cell diffuse, upper jaw bone radiography

## Abstract

A 47-year-old male with an unremarkable medical history was referred for atypical endodontic pain and treatment of his left upper molars. Clinical and radiographic examinations revealed an extensive, undefined osteolytic area around these teeth. A subsequent bone biopsy diagnosed diffuse large B-cell lymphoma, a high-grade non-Hodgkin’s lymphoma. The hematology team prescribed six cycles of chemotherapy, supplemented by two cycles of methotrexate. Practitioners should be alerted by atypical tooth pain to consider 3D imaging to exclude malignant pathology as early as possible.

*Teaching point:* An atypical tooth pain should alert the practitioner and guide them towards 3D imaging to eliminate diagnostic of malignant pathology as early as possible.

## Introduction

Over the past three decades, Cone Beam Computed Tomography (CBCT) has increasingly replaced conventional CT for 3D studies of teeth and maxillofacial structures. This digital imaging technique offers significant advantages in spatial resolution and dosimetry. CBCT is now a critical tool for dental surgeons in diagnosing oral pathologies and planning treatments, complementing clinical examinations and conventional 2D radiography [1–3].

## Case Report

A 47-year-old male presented for endodontic treatment of his left upper molars, with no medical history. He reported periodic discomfort and pain during mastication for two years, which worsened recently, along with temporary hypoesthesia of the left cheek. An emergency appointment two months earlier for left upper jaw pain was treated with antibiotics, but the pain recurred after a month.

**Figure 1:**
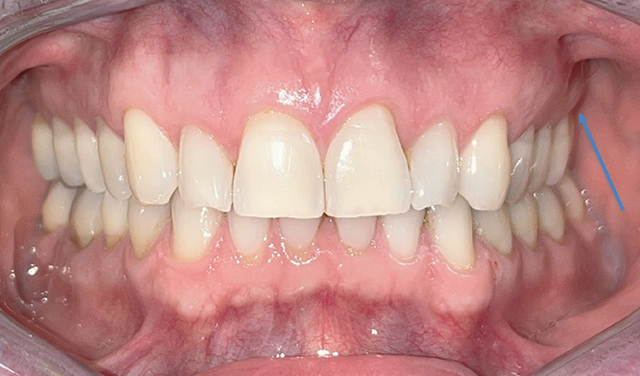
Intra-oral photography of the first endodontic consultation.

**Figure 2:**
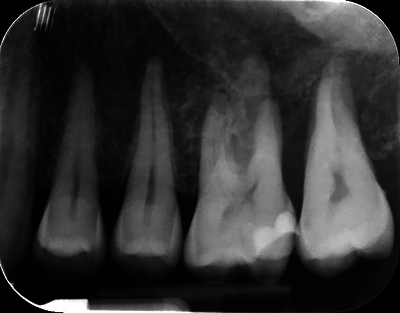
Retro-alveolar radiography at the first appointment.

**Figure 3:**
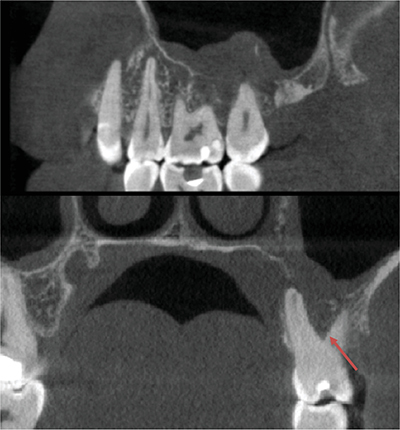
Computed tomography showing a large osteolytic area between left sinus and left upper molars (10 x10 cm (668 x 668 x 668)- 0,150 mm- 90 kV-8 mA 15,117s, ROMEXIS software).

Clinical examination revealed slight vestibular swelling adjacent to the first upper left premolar with healthy mucosa (Figure 1). The premolars and first upper left molar were painful upon percussion but had normal pulp vitality and no mobility. No cervical lymph nodes were palpable. A 2D periapical radiograph showed an atypical bone structure (Figure 2). A CBCT scan performed three months earlier revealed a poorly defined, large osteolytic area involving the left posterior maxillary bone walls and sinus floor (Figure 3).

**Figure 4:**
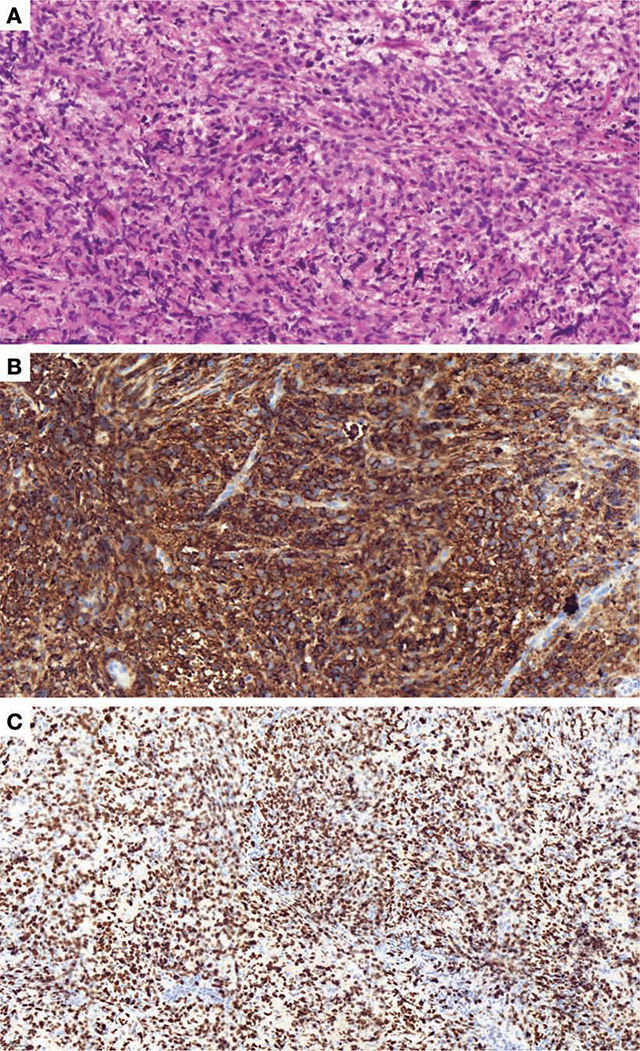
Histological analysis. A. A23 03155 HES_33.3x.: hematoxylin-eosin-safran (HES) coloration, original x33,3 magnification, tumor infiltration in sheets of non-cohesive cells, often crushed, of medium to large size, with barely visible cytoplasm, poorly nucleolated nucleus. Presence of apoptosis images. B. A23 03155 CD20_35 5x: immunohistochemical study using anti-CD20 antibody, original magnification x 35.5, diffuse positivity of all tumor cells in favor of proliferation of B lymphocytes C. A23 03155 KI67_17.6x: immunohistochemical study using the anti-KI67 antibody, original magnification x17.6, highlighting a high proliferation index, in favor of an aggressive B lymphoma.

**Figure 5:**
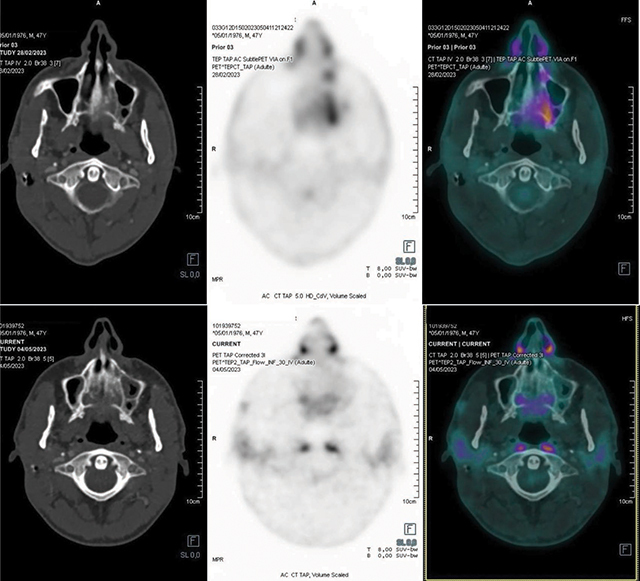
PET-SCAN after diagnosis and three months later after treatment.

A biopsy was performed, bone seemed moth-eaten, as friable tissue with a yellowish white appearance. Histological analysis showed bone infiltrated by large cells with scant cytoplasm and round nuclei (Figure 4), leading to a diagnosis of primary high-grade large B-cell non-Hodgkin’s lymphoma. A total body PET scan confirmed the maxilla as the sole pathology site. The patient underwent six courses of chemotherapy and two cycles of high-dose methotrexate to prevent cerebrospinal fluid relapse (Figure 5).

**Figure 6:**
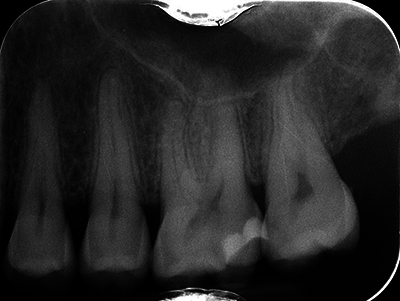
Radiography retro-alveolar post treatment (note the new densification around teeth).

After treatment, the patient reported no symptoms, and oral examination showed normal bone structure (Figure 6). Follow-up includes biannual oral examinations for the first year and annual checks for five years.

## Discussion

Oral lymphomas, accounting for 14% of head and neck malignancies [4], often present with symptoms mimicking odontogenic sources, complicating diagnosis [5]. Radiographic imaging, especially CBCT, is crucial for evaluating the extent of lytic areas [6]. Early biopsy and referral to an oral surgeon are essential for rapid diagnosis and treatment, significantly improving patient outcomes [7].

## Conclusion

This case emphasizes the importance of thorough analysis of atypical clinical symptoms and imaging findings. Rapid diagnosis and referral are critical for a favorable prognosis in cases of malignant oral pathologies.
